# Saunders’ Qestion Compends, No. 15

**Published:** 1891-01

**Authors:** 


SAUNDERS' QUESTION COMPENDS, No. 15. Essentials of the Diseases of Children; arranged in the form of Questions and Answers. Prepared especially for Students of Medicine. By WM. POWELL, M. D., Physician to the Clinic for Diseases of Children in the Hospital of the University of Pennsylvania, etc., etc. Philadelphia: W. B. Saunders. 1890.

This book, like all of its kind, has no proper excuse for its existence. The license to practise medicine should be granted only to those thoroughly qualified to obtain it, and all aids to obtain a medical degree without thorough information, in all branches of medical science, should be frowned down by all who are interested in a deeper, broader education, and a higher standard of educational qualification in aspirants for the degree of doctor of medicine. It is a pity to see instructors in medical schools countenancing such books, for the only purpose they serve is to aid a student to "cram" for examination. The superficial knowledge gained in this way is, of necessity, not lasting; and in medicine, more truly than in any other branch of science, "a little learning is a dangerous thing."

The book before us is neither better nor worse than others of the same class. The binding and letter-press are good. DeL. R.

